# Perceived social support and psychosocial adaptation in patients with chronic skin diseases: the mediating role of self-disclosure

**DOI:** 10.3389/fpsyt.2025.1731560

**Published:** 2026-02-18

**Authors:** Yue Zhu, Na Cai, Zhangyi Wang, Jiaxing Li, Tao Guo, Junchen He, Hong Wang, Jinjin Chen, Fengting Zhang, Shijie Huang, Yi Ren

**Affiliations:** 1 Tianjin Academy of Traditional Chinese Medicine Affiliated Hospital, Tianjin, China; 2 Clinical Medical Technology Demonstration Base For Emergency Treatment of Chest Pain in Hunan Province, The Affiliated Hengyang Hospital of Hunan Normal University & Hengyang Central Hospital, Hengyang, Hunan, China

**Keywords:** chronic skin disease, health psychology, perceived social support, psychodermatology, psychosocial adaptation, self-disclosure, stigma

## Abstract

**Objectives:**

To investigate the relationship between perceived social support, self-disclosure, and psychosocial adaptation among patients with chronic skin diseases, and examine the mediating role of self-disclosure within this relationship.

**Background:**

Chronic skin disease imposes not only physical burdens but also frequently precipitates significant psychosocial adaptation difficulties. However, current research remains insufficient in understanding the overall characteristics of psychosocial adaptation associated with such conditions and their key driving factors.

**Methods:**

A cross-sectional correlational design followed the Strengthening the Reporting of Observational Studies in Epidemiology checklist for quality reporting, with the findings of which being validated through a mediation model. Utilising convenience sampling, 317 patients with chronic skin disease were recruited from a total of eight Class A tertiary hospitals located within mainland China. Questionnaire surveys were conducted using a general information questionnaire, Perceived Social Support Scale, Distress Disclosure Index Scale, and Psychosocial Adaptation Scale. Data analysis utilised descriptive statistics, univariate analysis, Pearson correlation analysis, and a mediation effect model.

**Results:**

A total of 317 patients participated in the survey. Significant positive correlations were observed between psychosocial adaptation and the total scores for perceived social support and self-disclosure, as well as their respective dimensional scores (*r* = 0.703, *r* = 0.678, all *p* < 0.01). Mediational analysis indicated that self-disclosure partially mediated the relationship between perceived social support and psychosocial adaptation (Mediation was found to account for 17.8% of the total effect).

**Conclusion:**

The Results reports that the psychosocial adaptation scale, when benchmarked against the median mean score of the scale items, indicate an overall level that is moderately low. Perceived social support has the capacity to influence psychosocial adaptation by affecting the degree of self-disclosure provides a theoretical basis upon which subsequent development of targeted intervention measures can be grounded.

## 1 Introduction

Dermatosis refers to the pathological process caused by changes in the structure, morphology and function of all skin, including hair and nails, after being affected by many factors, and has various clinical manifestations (1). Chronic dermatosis refers to skin diseases with long course, slow progress, persistent or recurrent symptoms, and it is usually difficult to completely cure them (2). According to research, about 20% to 30% of the global population suffers from at least one chronic skin disease at different times, and chronic skin disease has become one of the diseases with high burden of non-fatal diseases in the world (3). Chronic skin disease is characterised by prolonged duration and a tendency to recur. The persistent itching, pain and cosmetic concerns it causes not only impose significant psychological distress and financial burden on patients, but also markedly impact their social interactions, occupational capacity and overall quality of life (4). Research shows that 30%-60% of patients with chronic skin diseases have psychological disorders related to diseases (4). According to a survey conducted by the British Association of Dermatologists, 3% of patients with dermatoses have primary mental disorders, 8% of patients with dermatoses have mental disorders that make their original mental problems worse, 14% of patients with dermatoses have mental disorders that worsen at the same time, 17% of patients with dermatoses need psychological support, and 85% of patients with dermatoses think that mental and social problems caused by dermatoses have become their main problems (5). With the occurrence and development of chronic skin diseases, on the one hand, patients need to bear economic pressure for treatment, on the other hand, their seriously damaged psychosocial function often leads to their inability to work normally. It has been reported that psoriasis can result in patients losing approximately 6.6% of their working hours each month on average (6). These data indicate that psychosocial maladjustment is highly prevalent among patients with chronic skin disease and constitutes a core burden. However, the specific psychosocial adaptation processes and underlying mechanisms remain poorly elucidated. Therefore, unravelling these mechanisms, comprehending patients’ holistic disease experience, and developing effective integrated mind-body care interventions are of paramount importance.

Psychosocial adaptation is a process in which individuals constantly adjust themselves to better adapt to the changes of surrounding environment and people and things in order to maintain the best physical and psychological state when facing stressors, including physical, psychological and social levels, and it is a process in which patients carry out systemic and active dynamic adjustment (7, 8). When patients with chronic diseases are in a stable state of mind and body and have goodpsychosocial adaptation, they can show high satisfaction with their physical and mental state and living conditions. When patients are not well adapted to psychological and social conditions, this may lead to various challenges in their daily lives, which could seriously compromise treatment efficacy and patient prognosis (9–11).

Perceived social support refers to the emotional experience and satisfaction of individuals who are in society and feel supported and respected by others (12).The research results of Nyman et al. (13) show that understanding social support can reduce patients’ anxiety and improve their quality of life. Other studies have shown that good social support can significantly enhance patients’ confidence in rehabilitation and promote positive adjustment (14). Based on the aforementioned theory and evidence, higher levels of perceived social support are theoretically associated with more positive emotional experiences, lower perceptions of treatment-related pain and fatigue, fewer experiences of social exclusion, and better psychosocial adaptation among patients with chronic skin disease.

Self-disclosure refers to the process that an individual reveals his true feelings, experiences and thoughts to others through various channels, which belongs to a coping style of the individual (15). It is found that individuals can express and pour out their negative emotions to others through self-disclosure, so as to relieve stress, and at the same time, focus on the future, actively cope with the pain caused by diseases and improve their quality of life (16). At the same time, self-disclosure can improve individual social support, alleviate negative emotions such as anxiety and depression, and reduce the negative impact of stressors on individuals (15). Based on the foregoing analysis, we hypothesise that effective self-disclosure is associated with positive stress adaptation, reduced negative emotions, diminished feelings of social isolation, and enhanced psychosocial adaptation among patients with chronic skin disease.

According to Roy adaptation model (17), we assume that chronic skin disease is the main stimulus faced by patients, and perceived social support as a cognitive system acts on self-disclosure, which in turn affects thepsychosocial adaptation level of patients.

Existing research has largely focused on the prevalence of psychosocial issues. However, within the complex mechanisms of psychosocial adaptation in chronic skin disease, the specific pathways through which social support and self-disclosure function as key psychosocial moderating factors remain insufficiently elucidated. Empirical exploration and theoretical integration concerning how patients can harness social support networks and engage in illness-related self-disclosure to foster positive adaptation and mitigate adverse effects warrant further investigation. Therefore, this study carried out a cross-sectional survey to evaluate the status quo of psychosocial adaptation of patients with chronic skin diseases and analyze the paths of social support and self-disclosure on psychosocial adaptation, so as to provide reference for medical staff to formulate intervention programs to promote the level of psychosocial adaptation of patients.

Notwithstanding the considerable attention afforded to the psychosocial adaptation of patients with chronic skin disease, it remains imperative to acknowledge the significant heterogeneity inherent within this group. diseases exhibit considerable variability with respect to symptom presentation, objective severity, duration of illness, subjective symptom burden (e.g. itching and pain) and psychological comorbidities (e.g. anxiety and depression). These factors are intertwined, exerting complex and profound influences on patients’ adaptation processes. The present study is chiefly concerned with psychosocial mechanisms, with particular reference to the possible function of perceived social support and self-disclosure in facilitating psychosocial adaptation.

### 1.1 Theoretical basis

In order to systematically elucidate the adaptive processes of patients with chronic skin disease under disease stress and to provide a theoretical framework for the core variables of this study, the Roy Adaptation Model is drawn upon. This model conceives the individual as an integrated adaptive system comprising biological, psychological, and social dimensions, with its core focus on coping with various stimuli and generating adaptive responses (17).

In the framework of this investigation, disease manifestations and their psychosocial consequences are conceptualised as primary sources of stress. The model posits the hypothesis that an individual’s cognitive and regulatory systems jointly process external stimuli. Specifically, perceived social support is conceptualised as part of the cognitive subsystem, involving an individual’s evaluation and interpretation of available support resources; whereas self-disclosure is regarded as a regulatory behaviour, constituting a patient’s active, adaptive response to external stimuli. The combined operation of these two systems ultimately influences and manifests in the individual’s adaptive response–namely, the level of psychosocial adaptation examined in this study.

**Figure 1:**
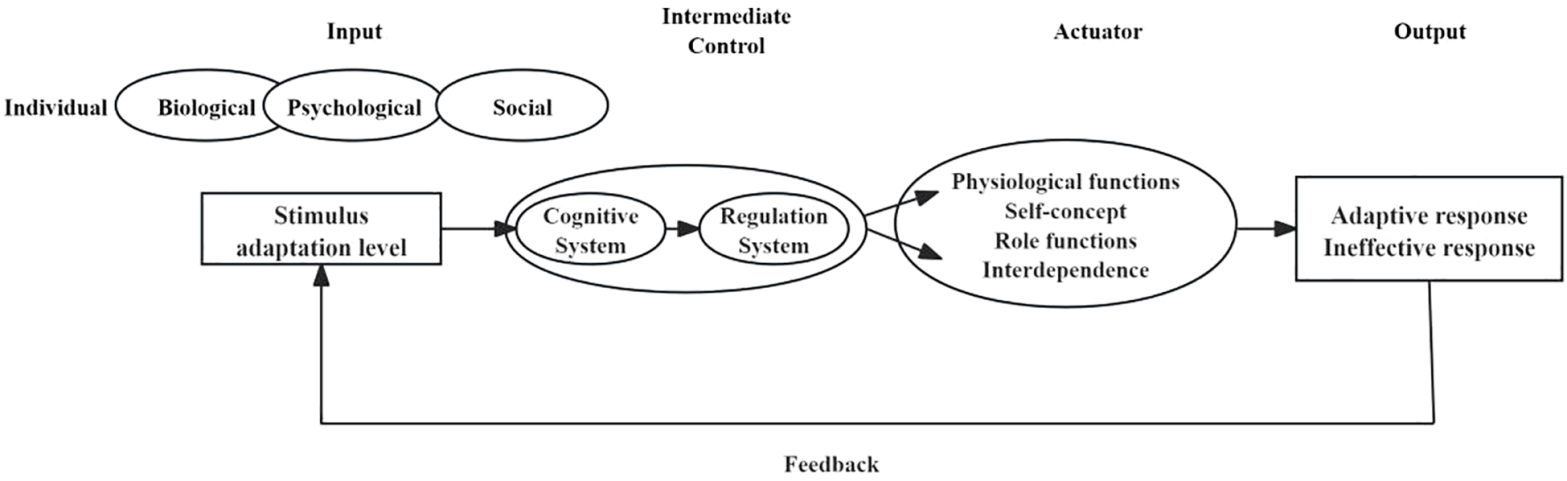
Theoretical basis.

It is therefore evident that Roy’s Adaptation Model provides a coherent theoretical pathway for the present study to explore how perceived social support (cognitive appraisal) and self-disclosure (regulatory behaviour) jointly influence psychosocial adaptation (adaptive response) among patients with chronic skin disease. The theoretical basis for this study is shown in **Figure 1**.

### 1.2 Study aim and hypotheses

Based on prior evidence, this study relying on Roy adaptation model, aims to analyze the correlation between perceived social support, self-disclosure and psychosocial adaptation of patients with chronic skin diseases, and assumes that self-disclosure plays an intermediary role between perceived social support and psychosocial adaptation. Based on this, we put forward the following research hypotheses:

Hypothesis 1: The higher the level of perceived social support, the higher the level of self-disclosure and psychosocial adaptation.
Hypothesis 2: The higher the level of self-disclosure, the higher the level of psychosocial adaptation.
Hypothesis 3: Self-disclosure plays a partial intermediary role between understanding perceived social support and psychosocial adaptation.

**Figure 2:**
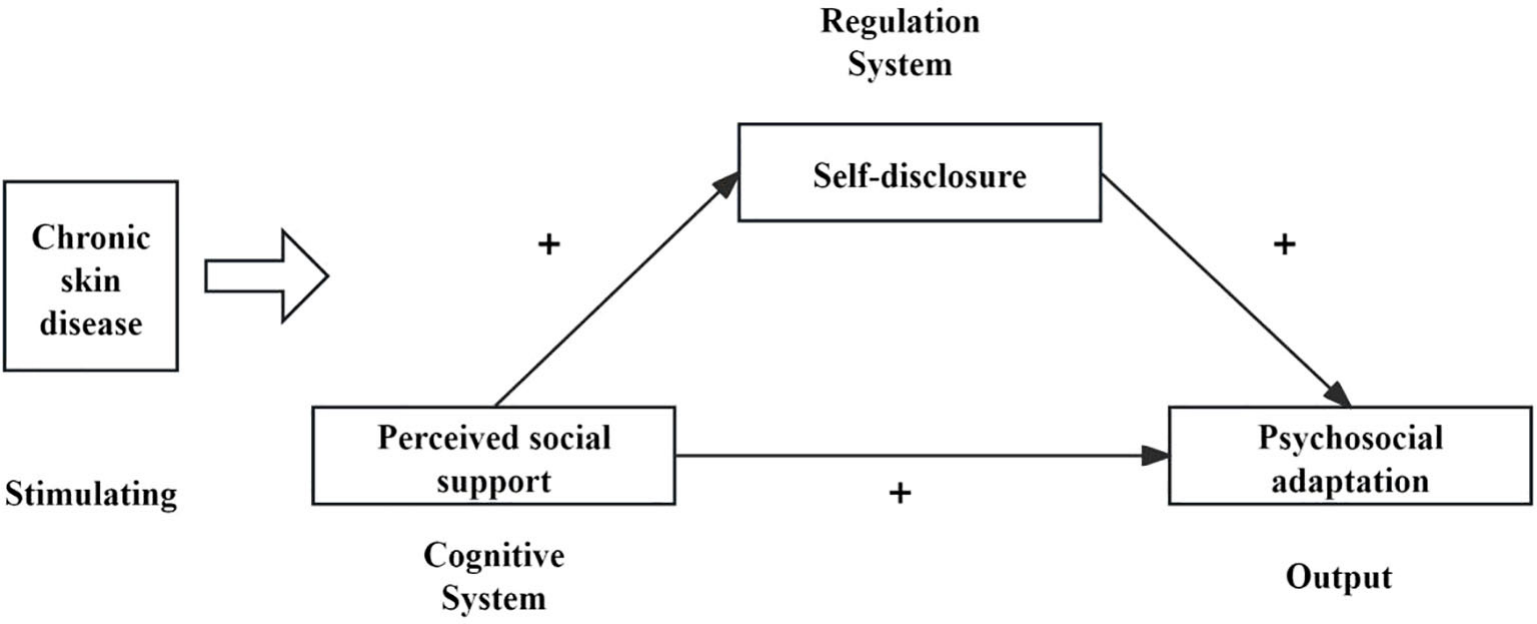
Hypothetical model.

The hypothetical model of this study is shown in **Figure 2**.

It is hypothesised that the results of this study will provide preliminary theoretical underpinnings and practical insights for the future development of nursing interventions designed to enhance the psychosocial adaptation of patients with chronic skin disease by optimising social support and promoting self-disclosure.

## 2 Methods

### 2.1 Study design

A cross-sectional observational study incorporating mediation analysis was adopted in this study. In order to ensure the full and complete report of the study, we adopted the STROBE for cross-sectional study (18).

### 2.2 Sample

Between March and July 2025, patients with chronic skin disease from eight Class A tertiary hospitals in mainland China were selected via convenience sampling. Inclusion criteria: (1) Age ≥ 18 years; (2) Diagnosed with a chronic skin condition (e.g., psoriasis, vitiligo, eczema) for over one month; (3) Possessed the basic communication and comprehension skills necessary for the completion of questionnaires either independently or with standardised, non-directive assistance provided by researchers; (4) Informed and voluntarily participating in the study. Exclusion criteria: (1) Severe cognitive or psychiatric impairment; (2) Concurrent serious medical conditions.

The efficacy analysis conducted in this study employs a multiple linear regression framework (fixed model) to test the significance of R² deviating from zero. In accordance with analogous studies, an effect size of f² = 0.15 was established. Under conditions of a significance level α= 0.05 and statistical power 1-β= 0.90, considering three independent variables, the sample size was determined using G*Power 3.1 software. The estimation indicated a minimum required sample size of 99 participants. This study finally collected 317 valid questionnaires, sufficient statistical power is ensured.

### 2.3 Measures

#### 2.3.1 Demographic characteristics

To control for potential confounding factors, sixteen general characteristics were included based on a literature review: gender, age, marital status, place of residence, housing conditions, educational attainment, method of payment for medical treatment, occupational status, type of skin disease, and location of skin lesions.

#### 2.3.2 Psychosocial adaptation scale for patients with chronic skin diseases

Compiled by Chinese scholar Zhang Xiujie (19), this 18-item scale encompasses three dimensions: emotional, self-perception, and social. Employing a five-point Likert scale, responses ranging from 0 to 4 correspond to “always”to “never”, yielding a total score between 0 and 72. Higher scores indicate better psychosocial adaptation among patients with chronic skin conditions. The Cronbach’s α coefficient of the scale in the present study was 0.943.

#### 2.3.3 Multidimensional scale of perceived social support

Developed by Zimet et al. (20) in 1988 and translated and revised by Jiang, Qianjin et al. (21) in 1999, this scale is suitable for diverse populations. It comprises three dimensions—family support, friend support, and other support—totalling 12 items. Employing a 7-point Likert scale, scores ranging from 1 (strongly disagree) to 7 (strongly agree) yield a total score between 12 and 84. Higher scores indicate greater perceived levels of social support. The Cronbach’s α coefficient of the scale in the present study was 0.970.

#### 2.3.4 Distress disclosure index

Developed by Kahn et al. (22) and adapted into Chinese by Li Xinmin (23) in 2009, this scale assesses the extent to which individuals express their true feelings to others. This single-dimensional scale comprises 12 items. It employs a 5-point Likert scale, where scores of 1 to 5 represent ‘Strongly disagree’ to ‘Strongly agree’, respectively. The total score ranges from 12 to 60, with higher scores indicating greater self-disclosure. The Cronbach’s α coefficient of the scale in the present study was 0.878.

### 2.4 Data collection

Prior to the investigation, consent was obtained from the management of a Grade A tertiary hospital. Between March and July 2025, questionnaires were distributed to chronic skin disease patients meeting inclusion and exclusion criteria via the Wenjuanxing online platform (Hunan Blue Star Information Technology Co., Ltd., Changsha, China). In order to ensure the anonymity of respondents and thereby encourage honest responses, the present survey instrument has been designed to be completed online and anonymously. Standardised instructions explained the study’s purpose, significance, and questionnaire completion method. Participants were informed they could withdraw at any time without repercussions, that participation was entirely voluntary, and that they would complete the questionnaire anonymously and independently based on informed consent.

### 2.5 Quality control

The questionnaire is configured such that all questions are mandatory to to minimise data gaps caused by omitted responses; technical restrictions shall be implemented to ensure that only one reply may be submitted per IP address, thereby preventing duplicate responses. Utilizing the response time distribution across all preliminary samples as a basis, submissions completed within three standard deviations below the average time are deemed “extremely rapid responses” and are excluded from consideration (24). In addition, consistency checks are performed on logically validated items within the questionnaire; submissions exhibiting obvious contradictions face similar exclusion.A total of 326 questionnaires were collected for this survey. After excluding 9 non-compliant responses, 317 valid questionnaires were obtained, representing an effective response rate of 97.24%.

### 2.6 Data analysis

In the quantitative analysis phase of this study, all data were analysed using SPSS 21.0 (IBM Corporation, Armonk, NY, USA) with the PROCESS macro (version 3.4.1) developed by Hayes for path analysis. Count data were described using frequencies and percentages. The Shapiro–Wilk test was utilised to evaluate the normality of distribution for all continuous variables. Continuous variables that satisfied the criteria of normality were described using the mean ± standard deviation (*M ± SD*), with intergroup comparisons being conducted via independent samples t-tests or analysis of variance (ANOVA). For non-normally distributed continuous variables, the data were described using the median and interquartile range (M (*P25, P75*)), with intergroup comparisons performed using non-parametric analysis of variance on the U statistic (Mann-Whitney U test) or the Kruskal-Wallis H test.Pearson correlation analysis explored associations between psychosocial adaptation and perceived social support, as well as self-disclosure. The PROCESS macro in SPSS software was employed to validate the mediating effect of self-disclosure between perceived social support and psychosocial adaptation. To control for potential confounding factors, regression analyses were conducted with perceived social support, self-disclosure, and psychosocial adaptation as respective dependent variables. Variables exhibiting significant correlations (*p* < 0.05) with at least one of the independent variables, mediating variables, or dependent variables were retained as candidate covariates. psychosocial adaptation as the dependent variable (Y), and self-disclosure as the mediating variable (M). These covariates were placed within the Covariates box, thereby ensuring that all path coefficients and final indirect effect estimates represented net effects after controlling for the influence of these variables.Effect sizes were assessed for significance by generating 95% confidence intervals via 5000 bootstrap samples. An effect was deemed statistically significant if the 95% confidence interval did not encompass zero. For two-tailed tests, differences were considered statistically significant when *p* < 0.05. It is important to note that, given the cross-sectional nature of the data, the mediation path analysis reported herein aims to reveal potential patterns of association between variables. It is not possible to infer causality or determine the direction of influence.

Prior to the commencement of parametric testing, a comprehensive assessment of the distributional characteristics of all continuous variables was undertaken. The skewness and kurtosis coefficients for each variable were found to fall within acceptable ranges, with absolute values below 1. No significant ceiling or floor effects were observed. These findings support the subsequent application of parametric statistical methods.

### 2.7 Ethical considerations

This study has been approved by the Medical Ethics Committee of the Affiliated Hospital of Tianjin Academy of Traditional Chinese Medicine(Ethics Review Numbe: LLKY2025-86). All participants signed informed consent forms. The research strictly adheres to the principles of the Declaration of Helsinki, ensuring subjects enjoy full rights and privacy protection throughout the study. All participants voluntarily signed informed consent forms to participate in the study and retained the right to withdraw at any stage. The research strictly adhered to principles of anonymity and confidentiality. None of the questionnaires included sensitive information such as names or national identification numbers; participants were identified solely by coded numbers. Researchers underwent specialised training and strictly observed confidentiality requirements, implementing data management measures to ensure participant information remained secure and was not disclosed.

## 3 Results

### 3.1 General information of patients with chronic skin disease

This study included 317 patients with chronic skin conditions, comprising 120 males (37.9%) and 197 females (62.1%); The youngest participant was 18 years old, while the oldest was 90 years old. The mean age was (47.42 ± 16.84) years. Seventy-three participants (23.0%) were aged <35 years, 167 (52.7%) were aged 35–<60 years, and 77 (24.3%) were aged ≥60 years. Additional demographic details are presented in **Table 1**.

**Table 1:** Descriptive and univariate analysis of psychosocial adaptation in patients with chronic skin diseases (*N* = 317, mean±SD).

Variables	*n*	%	Scores of psychosocial adaptation (mean±SD)	*t / F* value	*p-*value
Gender				2.132	0.034
Male	120	37.9	37.22±13.21		
Female	197	62.1	34.28±10.99		
Age (years)				11.699	<0.001
<35	73	23.0	36.47±8.90		
35∼<60	167	52.7	37.46±10.91		
≥60	77	24.3	29.89±14.72		
Marital status				19.444	<0.001
Married	212	66.9	37.95±10.17		
Unmarried	62	19.6	32.85±11.07		
Divorced or separated	8	2.5	40.25±5.04		
Widowed	35	11.0	23.31±15.84		
Educational attainment				205.272	<0.001
Primary school and below	66	20.8	17.20±9.44		
Lower secondary school	33	10.4	32.18±5.01		
Upper secondary school	83	26.2	37.10±1.49		
College diploma	22	6.9	41.64±5.54		
Bachelor's degree and above	113	35.6	44.50±6.68		
Place of residence				16.722	<0.001
Town/city	218	68.8	41.43±6.24		
Rural area	99	31.2	22.11±10.70		
Living arrangements				-2.198	0.030
Living alone	75	23.7	32.32±14.68		
Not living alone	242	76.3	36.35±10.82		
Average monthly household income (RMB)				35.208	<0.001
<3000	34	10.7	20.91±14.12		
3000-<5000	51	16.1	30.53±11.08		
5000-<8000	122	38.5	36.98±11.53		
≥8000	110	34.7	40.37±6.70		
Methods of payment for medical treatment				18.210	<0.001
Urban Employee Medical Insurance	244	77.0	37.89±10.19		
Rural Cooperative Medical Scheme	55	14.7	24.20±13.99		
Self-funded	6	1.9	33.00±5.44		
Private health insurance	6	1.9	35.17±9.85		
Other	6	1.9	39.00±4.15		
Exercise habits				33.837	<0.001
Regular exercise	97	30.6	40.61±6.94		
Irregular exercise	124	39.1	36.9±48.76		
No exercise	96	30.3	28.14±15.56		
Current employment status				2.423	0.016
In employment	93	29.3	37.60±9.32		
Not in employment	224	70.7	34.48±12.78		
Whether have any other chronic conditions				-10.576	<0.001
Yes	85	26.8	23.45±13.47		
No	232	73.2	39.77±7.57		
Type of skin condition				6.885	0.001
Infectious skin disease	140	44.2	36.21±8.77		
Autoimmune skin disease	69	21.8	30.84±11.73		
Allergic skin disease	108	34.1	37.24±14.70		
Skin lesion location				77.094	<0.001
Head and face	36	11.4	32.33±7.26		
Trunk	102	32.2	42.86±8.05		
Extremities	101	31.9	38.56±5.69		
Whole body	78	24.6	22.94±13.62		
Presence of itching in lesions				285.647	<0.001
Present	204	64.4	33.69±12.74		
Absent	113	35.6	38.47±9.66		
Current episode				11.664	<0.001
First episode	180	56.8	41.42±6.60		
Recurrence	137	43.2	27.47±12.76		
Duration (years)				28.382	<0.001
<6 months	189	59.6	39.08±8.45		
6 months to <1 year	34	10.7	37.62±12.99		
1 year to <5 years	51	16.1	28.82±11.55		
5 years to <10 years	31	9.8	20.35±13.79		
≥10 years	12	3.8	37.75±12.34		

Categorical variables were described with recourse to frequency (percentage), while continuous variables were described using the mean ± standard deviation. Comparisons between groups were performed using independent samples *t*-tests or one-way ANOVA.

### 3.2 Descriptive analysis of the level of psychosocial adaptation, perceived social support and self-disclosure

The total score of psychosocial Adaptation of 317 chronic skin disease patients was (35.39 ± 11.94), with an average item score of (1.97 ± 0.91). The total emotional dimension score was (16.24 ± 6.47), with an average item score of (2.03 ± 0.81). The total score for the self-perception dimension was (10.31 ± 5.00) points, with an average item score of (1.72 ± 0.83) points; The total score for the social dimension was (8.85 ± 3.22), with an average item score of (2.21 ± 0.81). The total score for perceived social support was (42.81 ± 13.72), and the total score for self-disclosure was (35.43 ± 9.18). As shown in **Table 2**.

**Table 2:** The scores of psychosocial adaptation, perceived social support and self-disclosure among patients with chronic skin diseases (*N* = 317, mean±SD).

Item	Dimensional score	Average of entries	Ranking
Psychosocial adaptation total score	35.39±11.94	1.97±0.91	—
Emotions	16.24±6.47	2.03±0.81	2
Self-awareness	10.31±5.00	1.72±0.83	3
Society	8.85±3.22	2.21±0.81	1
Perceived social support total score	42.81±13.72	3.57±1.14	—
Family support	14.98±4.80	3.75±1.20	1
Friends support	13.57±4.70	3.39±1.78	3
Other support	14.26±4.88	3.56±1.22	2
Self-disclosure total score	35.43±9.18	2.95±0.76	—

The rankings are derived from the mean score of the entries across the full range of each variable's dimensions.

### 3.3 Correlational analysis of psychosocial adaptation, perceived social support, and self-disclosure in patients with chronic skin diseases

Pearson correlation analysis revealed that psychosocial adaptation among patients with chronic skin diseases was positively correlated with total scores and individual dimension scores of perceived social support (*r* = 0.398-0.722, *p<*0.01). Perceived social support was positively correlated with total scores and individual dimension scores of self-disclosure (*r* = 0.520-0.602, *p<* 0.01). Self-disclosure among patients with chronic skin disease was positively correlated with both the total score and all subscale scores of psychosocial adaptation (*r* = 0.451-0.678, *p<* 0.01). Detailed results of the correlation analysis between perceived social support, self-disclosure, and psychosocial adaptation among patients with chronic skin disease are presented in **Table 3**. It is important to note that all sub-dimensions of social support demonstrated high correlations with each other and with the overall psychosocial adaptation score (*r* > 0.60). In order to examine potential multicollinearity issues, we calculated the variance inflation factors (VIF) for all predictor variables. The results of the analysis revealed that the VIF values exhibited a range between 4.184 and 6.194 across all primary analysis models. For instance, the VIF values were found to be 4.184 in the Family Support dimension, 6.194 in the Friends Support dimension, and 6.074 in the Other Support dimension. It was observed that all these values fell below the critical threshold of 10. This indicates that, despite strong inter-construct correlations, multicollinearity did not reach levels that would significantly distort parameter estimates.

**Table 3:** The correlation between psychosocial adaptation, perceived social support and self-disclosure among patients with chronic skin diseases (*N* = 317, *r*).

Dimensions	Perceived social support total score	Family support	Friends support	Other support	Self-disclosure total score	Psychosocial adaptation total score	Emotions	Self-awareness	Society
Perceived social support total score	1								
Family support	0.942**	1							
Friends support	0.964**	0.860**	1						
Other support	0.957**	0.838**	0.901**	1					
Self-disclosure total score	0.595**	0.520**	0.580**	0.602**	1				
Psychosocial adaptation total score	0.703**	0.615**	0.676**	0.722**	0.678**	1			
Emotions	0.639**	0.556**	0.611**	0.663**	0.627**	0.897**	1		
Self-awareness	0.448**	0.398**	0.431**	0.454**	0.451**	0.657**	0.277**	1	
Society	0.626**	0.543**	0.610**	0.639**	0.552**	0.886**	0.886**	0.326**	1

**p<0.01.

### 3.4 The mediating effect of self-disclosure in chronic skin disease patients on the relationship between perceived social support and psychosocial adaptation

**Figure 3:**
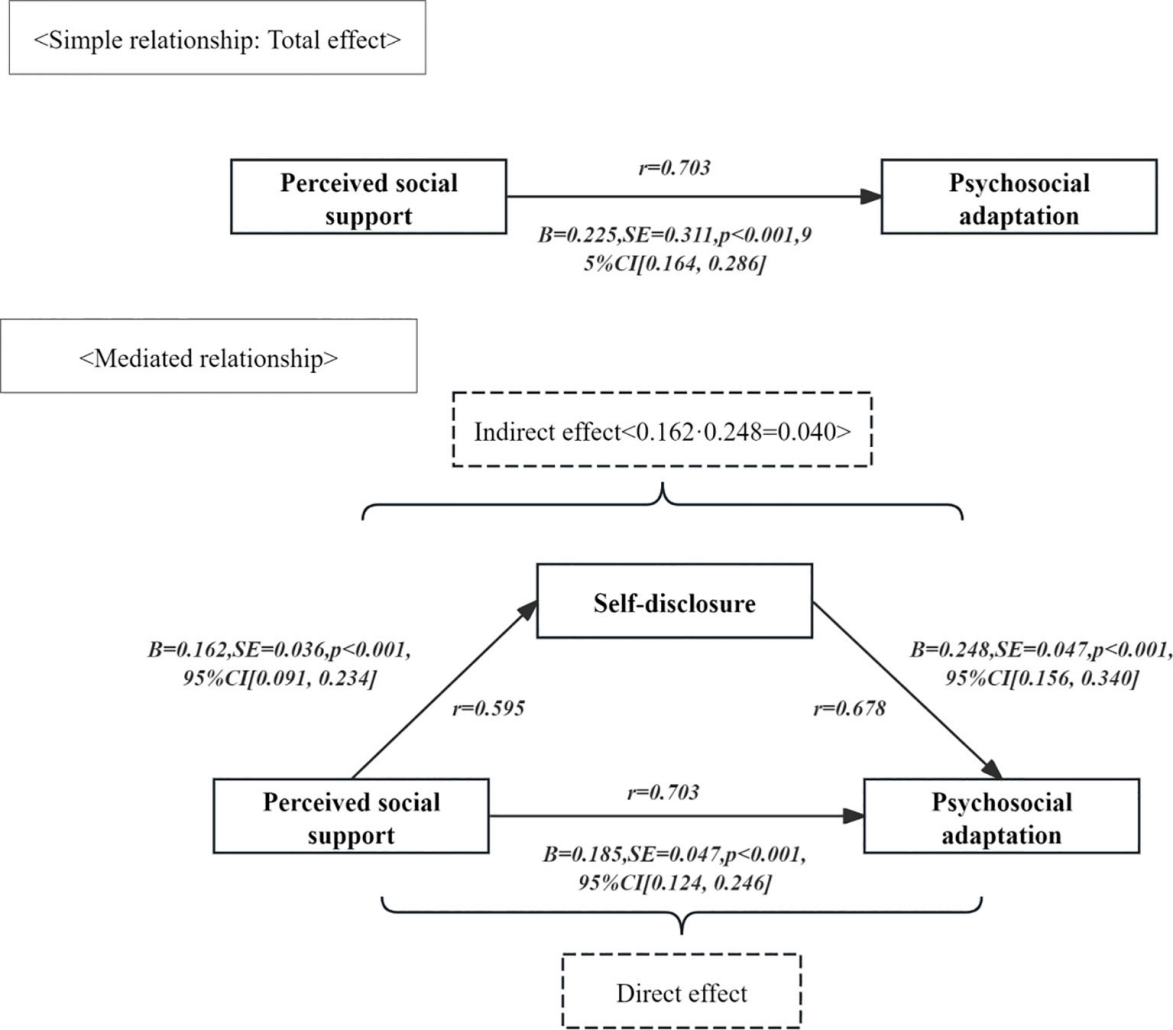
The direct, indirect, and total effects between psychosocial adaptation, perceived social support, and self-disclosure.

Prior to running the formal mediation model, we conducted regression analysis to retain variables exhibiting significant correlations (*p* < 0.05) with at least one of the independent variables, mediating variables, or dependent variables as candidate covariates. The finalised covariates were: average monthly household income, marital status, educational attainment, exercise habits, place of residence, presence of other chronic conditions, type of skin disease, and location of skin lesions.The mediating effect was examined using the Process function (Model 4) in SPSS software. The independent variable was perceived social support among patients with chronic skin disease, the mediating variable was self-disclosure, and the dependent variable was psychosocial adaptation, incorporating the aforementioned covariates. A Bootstrap test employing 5, 000 resamples was conducted. Results indicated that both the direct effect of perceived social support on psychosocial adaptation and the upper and lower bounds of the 95% confidence interval for self-disclosure’s mediating effect (based on 5, 000 Bootstrap samples) excluded zero. The total effect value of perceived social support on psychosocial adaptation was 0.225 (*p <* 0.01, 95% *CI* 0.164~0.286). Specifically, the direct coefficient of perceived social support on psychosocial adaptation was 0.185 (*p <* 0.01, 95% *CI* 0.124 - 0.246). The indirect effect of self-disclosure on psychosocial adaptation was calculated as 0.162 × 0.248 = 0.040, accounting for 17.8% of the total effect value of 0.225 (*p <* 0.01). As shown in **Table 4** and **Figure 3** for details. This model is responsible for a significant percentage of the variability in psychosocial adaptation. However, given the cross-sectional nature of the data, this high value primarily reflects the model’s fit to the sample data, and therefore, caution should be exercised when extrapolating to causal predictions.

**Table 4:** The mediating effect of self-disclosure between perceived social support and psychosocial adaptation among patients with chronic skin diseases (N=317).

Model Path	*B*-value	Standard error	*t-*value	*p-*value	*F*	*R-*value	*R^2^-*value	95% CI
Total effect					134.024	0.893	0.797	
Perceived social support→Psychosocial adaptation	0.225	0.311	7.233	<0.001				[0.164, 0.286]
Direct effects					38.298	0.727	0.529	
Perceived social support→Self-disclosure	0.162	0.036	4.453	<0.001				[0.091, 0.234]
Perceived social support→Psychosocial adaptation	0.185	0.031	6.007	<0.001				[0.124, 0.246]
Self-disclosure→Psychosocial adaptation	0.248	0.047	5.304	<0.001				[0.156, 0.340]
Indirect effects					—	—	—	
Perceived social support→Self-disclosure→Psychosocial adaptation	0.040	0.012	—	—				[0.019, 0.067]

## 4 Discussion

This study aimed to verify whether self-disclosure mediates the relationship between perceived social support and psychosocial adaptation among patients with chronic skin conditions. Findings indicate that self-disclosure partially mediates the positive correlation between perceived social support and psychosocial adaptation. This research provides scientific grounds for developing targeted interventions, while offering significant insights into enhancing self-disclosure abilities and promoting psychosocial adaptation among chronic skin disease patients.

### 4.1 Levels of psychosocial adaptation, perceived social support and self-disclosure in patients with chronic skin diseases

The findings of this study indicate that the 317 patients with chronic skin conditions achieved an overall psychosocial adaptation score of (35.58 ± 11.88), with an average item score of (1.98 ± 0.66). Compared to the median item score, this overall level is classified as moderately low. Psychosocial adaptation among patients with chronic skin conditions encompasses three dimensions: emotional, self-perception, and social. The highest mean score was recorded for the “social” dimension, indicating that patients’ family and friends did not avoid contact or treat them differently due to their skin condition. The lowest mean score was observed in the “self-perception” dimension, suggests that skin conditions significantly impact patients’ self-esteem and body image. Afflicted by visible symptoms, patients express dissatisfaction with their appearance and capabilities, harbouring doubts about their ability to resume pre-illness work and lifestyle. Potential contributing factors include: (1) Patients with chronic skin disease often present with highly visible, difficult-to-conceal lesions such as erythema and desquamation. This renders them subjects of curiosity, misunderstanding, or even discrimination from others, leading to appearance anxiety. This reluctance to engage socially impedes the fulfilment of social functions and the acquisition of positive feedback, creating a vicious cycle; (2) A review of the literature indicatest that the unpredictable nature of disease recurrence and treatment outcomes fosters persistent feelings of powerlessness and a crisis of trust in one’s own body. This drains patients’ energy, undermines confidence in recovery, hinders acceptance of their current selves, and may adversely affects self-esteem and body image (25). (3) It is posited that this psychological distress, arising from altered appearance and treatment uncertainty, may be rooted in a more fundamental disturbance of self-perception. Abdi et al. recently put forward the hypothesis that persistent somatic symptoms in chronic skin disease, in conjunction with visible lesions, have the potential to erode the clear distinction between bodily boundaries and self-identity experienced by patients (26). When skin issues persistently serve as a source of distress and focal point of attention, individuals may encounter difficulties in separating the “affected skin” from the “whole self”. This loss of body-self coordination may pose profound challenges to emotional regulation and interpersonal functioning. Drawing upon the aforementioned analysis, the present study puts forward possible avenues for future targeted interventions: Subsequent research might investigate the development of integrated support programmes, including: Firstly, the combination of skin lesion management with cognitive behavioural interventions (e.g., Acceptance and Commitment Therapy) to assist patients in alleviating appearance anxiety and reconstructing positive self-perception; Secondly, the optimisation of disease understanding and communication patterns among patients and their support systems through public health education, thereby providing more empathetic emotional support; Thirdly, fostering a respectful, non-discriminatory supportive environment in clinical and community settings, whilst attempting to introduce structured peer support groups to reduce loneliness and stigma, is of paramount importance. Fourthly, assisting patients in managing treatment burdens through empowerment education and management, enabling them to find meaning within their disease journey and thereby promoting overall psychosocial adaptation, is of equal importance. These potential directions warrant systematic development and efficacy validation through future interventional studies.

The findings of this study indicate that the total perceived social support score among 317 patients with chronic skin conditions was (42.81 ± 13.72) points, with an average item score of (3.57 ± 1.14) points. Compared to the median item score, the overall level was moderately low, consistent with the findings of Siddiqui et al. (27). Patients with chronic skin conditions perceive social support across three dimensions: family support, peer support, and other forms of support. Among these, the highest average score was recorded for the “family support” dimension, while the lowest average score was for the “peer support” dimension. This indicates that patients receive greater support from family than from friends. Possible reasons include: (1) Persistent itching, pain, and discomfort can lead to irritability and reduced energy, making it difficult to maintain focus and enjoyment during social activities. Patients may consequently decline invitations from friends, leading to reduced interaction and, consequently, diminished support from this source; (2) Family support tends to be more tangible and routine (e.g., assisting with medication application, sharing household responsibilities, accompanying to medical appointments), whereas friends may be uncertain how to offer substantive assistance, often limiting their support to verbal inquiries, which may be perceived as less substantial (28). It is posited that, in view of these findings, future health management practices may focus on enhancing, in a systematic manner, patients’ perceptions of support and of utilisation efficiency. Potential avenues for exploration include the following: the empowerment of patients through educational initiatives to optimise the responsiveness and support skills within their care networks (particularly peer networks); the employment of psychological interventions to mitigate internalised barriers such as stigma that impede the uptake of support; and attempts to disrupt the potential vicious cycle of “illness – stigma – low social support” by establishing integrated multi-support networks involving family, fellow patients, and professional teams. This approach may, thereby, offer pathways to improve patients’ overall health outcomes.

The findings of this study indicate that the total self-disclosure scores of 317 chronic skin disease patients were (35.43 ± 9.18) points, with an average score per item of (2.95 ± 0.76) points. Overall, these scores were at a moderate level, consistent with the results of Fang et al.’sstudy on cancer patients (29). This finding may be explored from the socio-psychological perspective of chronic skin disease. The existing literature reveals that visible lesions associated with the disease are frequently linked to patients’ feelings of inferiority and shame. Individuals may internalise societal prejudices, fearing negative judgements, which consequently impedes their willingness to engage in self-disclosure (30). At the same time, they worry that discussing the distress of illness, the financial burden of treatment, and the uncertainty of the future may place pressure and strain upon loved ones, or disrupt existing harmonious relationships, fearing they might become a “troublesome” patient. Consequently, they are prone to adopt a protective silence. Further exploration is warranted into the profound influence that patients’ underlying psychological structures exert on their self-disclosure behaviours. Research indicates that an individual’s attachment style and defence mechanisms are pivotal factors in shaping their coping mechanisms with psychological distress and interpersonal interaction patterns (31). Insecure attachment patterns and maladaptive defence mechanisms (such as denial or repression) impede open, constructive emotional expression under stress. In contrast, secure attachment and well-developed defence mechanisms (such as sublimation or humour) enable the development of healthier interpersonal boundaries and communication patterns, consequently, the disclosure dilemma experienced by patients with chronic skin disease stems not solely from fears of stigmatisation and relationship issues, but may also be indicative of their underlying attachment patterns and emotional regulation strategies (31). Research indicates that self-disclosure can enhance an individual’s self-understanding, promote mental wellbeing, and is a key factor in improving patients’ quality of life (32). Therefore, the findings of this study imply that in the management of chronic skin disease, focus should be given to patients’ willingness and ability for self-disclosure, and this should be prioritised. Future supportive interventions may include the following: firstly, the alleviation of patients’ stigma and fear of expression through psychoeducation and cognitive interventions; secondly, the enhancement of their confidence in self-disclosure through communication skills training, whilst optimising the responsiveness of their support systems. In addition, clinical interventions may draw upon attachment theory and psychodynamic perspectives in order to explore methods of assisting patients in identifying and modifying their insecure internal working patterns and maladaptive defence mechanisms. This approach aims to fundamentally enhance patients’ psychological capacity for adaptive self-disclosure. The aforementioned interventions, if implemented, would create a safer, more expressive environment for patients, thereby potentially supporting improvements in their psychosocial adaptation.

### 4.2 The correlations of perceived social support, self-disclosure and psychosocial adaptation

The findings of this study indicate that psychosocial adaptation among patients with chronic skin diseases exhibits a positive correlation with both the total score and individual dimension scores of perceived social support (*r* = 0.703, *p <* 0.01). That is, higher levels of perceived social support correlate with greater psychosocial adaptation among these patients, consistent with the findings of Xiong et al. (33) in their study of patients with acute myocardial infarction. Perceived social support reflects the tangible material and psychological assistance individuals receive through their social networks can influence patients’ subjective attitudes, playing a crucial role in their engagement with treatment. It serves as a reliable safeguard for patient prognosis and clinical outcomes (12). Higher levels of social support have been found to correspond with reduced psychological burden in patients, as well as increased engagement in self-management. These factors may therefore constitute a potential pathway associated with improved disease control and psychosocial adaptation. Conversely, On the other hand, patients who do not receive sufficient social support face the challenge of managing their own treatment while also attending to their daily living needs. This dual responsibility, known as the “treatment burden”, has been shown to be associated with poorer disease control and reduced social reintegration (34). It is therefore proposed that future clinical practice and research should systematically focus on patients’ social support systems. Following a comprehensive assessment of support availability, for example, the development of interventions aimed at enhancing the perception and utilisation of support could be pursued. Such measures might include optimising family communication, establishing structured peer support networks, and integrating self-management training with psychological counselling. This approach seeks to provide a more integrated support framework for improving patients’ psychosocial adaptation. This offers a potential avenue for refining chronic skin disease management models from a psychosocial perspective.

The findings of this study indicate that psychosocial adaptation among patients with chronic skin conditions exhibits a positive correlation with both the total self-disclosure score and scores across its individual dimensions (*r* = 0.678, *p <* 0.01). That is, higher levels of self-disclosure correlate with greater psychosocial adaptation among these patients, consistent with the findings of Pan Zhixia et al. in their study of breast cancer patients (35). Self-disclosure is the process whereby an individual reveals information about their emotions, views, experiences, and feelings to others (15). A review of the relevant literature leads to the hypothesis that higher levels of self-disclosure correlate with reduced psychological distress and more positive mental states. Articulating illness-related thoughts and feelings enables patients to engage in emotional catharsis and cognitive processing. Concurrently, proactively sharing health information facilitates healthcare teams and loved ones in more accurately understanding their needs, thereby potentially enabling more tailored support. These processes are collectively associated with alleviating social isolation and promoting social integration (36). Consequently, the findings of this study suggest that focusing on and facilitating patients’ self-disclosure may be significant in the management of chronic skin disease. Future supportive interventions may explore how to systematically cultivate environments that encourage expression, for instance through developing secure communication channels and training healthcare professionals and family members in active listening and responsive techniques. It is hypothesised that this may assist patients in overcoming barriers to disclosure and observe whether such approaches yield positive effects on improving their psychosocial adaptation.

### 4.3 Mediating role of self-disclosure on perceived social support and psychosocial adaptation

Based on cross-sectional data this study reveals a mediating pathway for self-disclosure between perceived social support and psychosocial adaptation, with the mediating effect accounting for 17.8% (*p* < 0.01), thus supporting a latent associative pattern. Theoretical considerations suggest a congruence between the statistical relationship under consideration and Roy’s adaptation model (17). The aforementioned model posits that when individuals encounter stressors (chronic skin disease), their cognitive systems (perceptions of support) influence adaptation outcomes through regulatory behaviours (self-disclosure). The data patterns that have been observed in this study are found to conform to this theoretical framework: namely, that higher perceived social support may foster a psychological environment of safety and acceptance for patients, thereby facilitating more open self-disclosure. Self-disclosure, as a moderating behaviour, may then be associated with better psychosocial adaptation through pathways such as promoting emotional catharsis, gaining understanding, and obtaining targeted support, this is also consistent with the findings of relevant literature studies in this field (37). The pathways revealed in this study may also be further theoretically elucidated through the dynamic model of psychological resilience. As Rossi et al. emphasise, psychological resilience is not a static trait, but rather a dynamic product of the interaction between individuals and situational protective factors during risk coping processes (38). In the framework of this study, perceived social support is regarded as a pivotal contextual protective resource, while positive self-disclosure is considered an individual’s capacity to proactively mobilise this resource for cognitive-emotional regulation. The effective integration of these two elements collectively constitutes a resilient process that fosters positive adaptation among chronic disease patients. This perspective incorporates the empirical findings into broader positive and developmental psychology frameworks, thereby underscoring the critical importance of cultivating adaptive interpersonal interactions during periods of adversity for the development of psychological resilience. In conclusion, this study has confirmed the presence of substantial statistical correlations and mediating pathways among social support, self-disclosure, and psychosocial adaptation. This finding indicates that when examining and facilitating psychosocial adaptation in patients with chronic skin disease, the interactive relationship between social support systems and individual expressive behaviours should be given due consideration. It is therefore posited that future intervention studies should explore integrated support programmes that simultaneously enhance patients’ perceived social support and prioritise the creation of safe conditions for self-expression. As an alternative approach, the enhancement of individuals’ intrinsic expressive skills (personal resilience components) in conjunction with the optimisation of their external supportive interpersonal environment (situational resilience components) is proposed. This synergistic approach is intended to promote adaptation.

This essay seeks to examine the factors that have led to the present situation, in which there is a high prevalence of the disease in the population. It will firstly review the literature on the subject, in order to establish which factors have been identified by previous research. It will then proceed to analyse the data in order to identify any trends or patterns that may be indicative of the causes of the disease. Finally, it will offer a conclusion based on the findings of its analysis and a discussion of the implications of these findings.The objective of such programmes should be to achieve a more comprehensive improvement in patients’ psychosocial adaptation levels.

In summation, the present study, which is grounded in Roy’s adaptation model, demonstrates from the perspectives of perceived social support and self-disclosure that both factors exhibit a strong statistical association with psychosocial adaptation in patients with chronic skin disease, along with mediating pathways. This finding provides new empirical evidence for understanding the adaptive mechanisms within this population. A review of the literature reveals that intervention studies targeting psychosocial adaptation in patients with chronic skin disease predominantly focus on fields such as cancer, with relatively insufficient attention directed towards dermatology patients. Furthermore, a close examination of existing interventions reveals significant variations and limitations in content, methodology, and outcome assessment. The findings of this study would appear to indicate that when developing future intervention programmes, social support and self-disclosure should be prioritised as core targets. For instance, it would be advisable for healthcare institutions and administrative bodies to consider drawing upon the relational pathways revealed in this study in order to explore the design of integrated support programmes. By systematically enhancing patients’ perceived support and sense of security in self-expression, it would be possible to observe whether such approaches can effectively promote patients’ psychosocial adaptation. This would then be able to provide more targeted, evidence-based guidance for refining the comprehensive physical and mental management of chronic skin diseases.

### 4.4 Limitations and strengthens

This study analysed the relationship between perceived social support, self-disclosure, and psychosocial adaptation among patients with chronic skin conditions, and explored the mediating pathways involved, offering an innovative perspective for the care of such patients. However, this study has several limitations: (1) The sample was restricted to patients with chronic skin disease from eight Grade A tertiary hospitals in mainland China. The sample size was small and the sampling method was simple, may not be entirely representative of the broader population of patients with chronic skin disease, particularly those with milder conditions, those not attending tertiary hospitals, or those at the extremes of the age distribution. This could potentially affect the generalisability of the findings. Future studies are advised to employ representative sampling strategies and expand the geographical scope of the sample to enhance the generalisability of the findings; (2) The cross-sectional design precludes accurate determination of causal relationships and directionality among perceived social support, self-disclosure, and psychosocial adaptation. Future large-scale, multi-level, multi-centre studies incorporating longitudinal and qualitative research are warranted to validate findings; (3) Self-reported data based on subjective perceptions are susceptible to bias. Subsequent studies should incorporate multi-dimensional perspectives, including evaluations of psychosocial adaptation by family members and healthcare providers of patients with chronic skin disease. (4) The indicators of dermatological severity incorporated within this study were insufficiently comprehensive, potentially constraining the in-depth examination of the complex interactions between psychosocial variables and disease characteristics; (5) The collection of all data was conducted through the administration of self-report questionnaires during single interviews. This methodological approach may potentially result in common method bias and introduce the possibility of response bias, such as social desirability, and the questionnaire has been designed to require responses in order to minimise the occurrence of missing data may potentially compel some participants to make arbitrary choices when uncertain, which could exert a subtle influence on the depth and accuracy of the data.

## 5 Conclusion

The present study has yielded findings that suggest the psychosocial adaptation levels of patients with chronic skin disease necessitate enhancement. A significant positive correlation has been identified between perceived social support and self-disclosure, with self-disclosure assuming a partial mediating role. These findings contribute to the body of empirical evidence that elucidates the psychosocial adaptation mechanisms operative within this demographic.

It may be proposed that future clinical practice and research prioritise the following directions. The first direction would be the systematic assessment of patients’ psychosocial adaptation within healthcare settings. The second would be the active exploration of effective methods for fostering safe, supportive therapeutic environments. The third would be the attempt to construct an intervention framework integrating social support with self-disclosure facilitation. Through these endeavours, new potential pathways may emerge for the enhancement of psychosocial adaptation, the promotion of physical and mental recovery, and the improvement of quality of life among patients with chronic skin disease.

## 6 Relevance to clinical practice

In this study, patients with chronic skin conditions exhibited psychosocial adaptation at a moderately low level, highlighting the urgent need for healthcare institutions and community health services to prioritise the quality of life and mental wellbeing of this population. Furthermore, the research identified that the association between perceived social support and psychosocial adaptation is partially mediated by self-disclosure. Consequently, incorporating self-disclosure promotion strategies into holistic health management programmes for chronic skin conditions holds significant reference value.

Integrating structured self-disclosure interventions with supportive environmental development into clinical follow-ups and health guidance not only enhances patients’ utilisation of social support and levels of self-disclosure, but also effectively improves their psychosocial adaptation, alleviates stigma associated with illness, and boosts confidence in long-term disease management. Moreover, facilitating patient self-disclosure significantly improves the quality of doctor-patient communication and treatment adherence, while enhanced psychosocial adaptation helps slow disease progression and reduce the risk of comorbidities. This study offers new pathways for chronic disease management and psychological intervention practices, providing evidence-based guidance and innovative directions for improving the overall health outcomes of patients with chronic skin disease.

## 7 Public health and policy implications

It is imperative to acknowledge that psychosocial adaptation is situated within a comprehensive framework of social structures when interpreting and implementing the results of this study. As Polaskey et al. highlight, structural elements such as social inequality, economic constraints, and access to healthcare resources profoundly restrict disease management and quality of life among patients with chronic skin disease (39). It can therefore be posited that interventions aimed at improving psychosocial adaptation through enhanced social support and facilitated self-disclosure have the potential to yield more profound and equitable public health impacts, when integrated with macro-level efforts to reduce health inequalities and improve healthcare policies. This area merits further research, adopting an interdisciplinary, multi-level perspective.

## Data Availability

The raw data supporting the conclusions of this article will be made available by the authors, without undue reservation.
